# Supplementary material for: Effectiveness of Following Mediterranean Diet Recommendations in the Real World in the Incidence of Gestational Diabetes Mellitus (GDM) and Adverse Maternal-Foetal Outcomes: A Prospective, Universal, Interventional Study with a Single Group. The St Carlos Study
Source: Nutrients. 2019 May 28;11(6):1210. doi: 10.3390/nu11061210 (PMC6627921; doi:10.3390/nu11061210)
Supplement: Supplementary File 1 [file nutrients-11-01210-s001.pdf]

METADATA

```
GET
  FILE='C:\Users\acall\Desktop\Repositorio\DEIDENTIFIED_REALWORDL.sav'.
DATASET NAME Conjunto_de_datos1 WINDOW=FRONT.
USE ALL.
COMPUTE filter_$=(GDM_DIAGNOSIS = 2).
VARIABLE LABELS filter_$ 'GDM_DIAGNOSIS = 2 (FILTER)'.
VALUE LABELS filter_$ 0 'Not Selected' 1 'Selected'.
FORMATS filter_$ (f1.0).
FILTER BY filter_$.
EXECUTE.
FREQUENCIES VARIABLES=ETHNICITY
  /ORDER=ANALYSIS.
```

ETHNICITY

		Frecuencia	Porcentaje	Porcentaje válido	Porcentaje acumulado
Válidos	caucasica /incluye española	574	61,6	61,6	61,6
	Eslava Rumania-Bulgaria...	48	5,2	5,2	66,7
	Hispanica	284	30,5	30,5	97,2
	china	9	1,0	1,0	98,2
	africana	14	1,5	1,5	99,7
	otras	3	,3	,3	100,0
	Total	932	100,0	100,0	

FAMILY_HISTORY

		Frecuencia	Porcentaje	Porcentaje válido	Porcentaje acumulado
Válidos	no existen	246	26,4	26,4	26,4
	diabetes	45	4,8	4,8	31,3
	HTA	56	6,0	6,0	37,3
	dislipemia	84	9,0	9,0	46,3
	obesidad	108	11,6	11,6	57,9
	2 situaciones	215	23,1	23,1	81,0
	mas de 2	174	18,7	18,7	99,7
	7	1	,1	,1	99,8
	desconocido	2	,2	,2	100,0
	Total	931	99,9	100,0	
Perdidos	Sistema	1	,1		
Total		932	100,0		

GESTATIONAL_HISTORY

		Frecuencia	Porcentaje	Porcentaje válido	Porcentaje acumulado
Válidos	ninguno	551	59,1	59,1	59,1
	aborto	311	33,4	33,4	92,5
	GDM	26	2,8	2,8	95,3
	HTA	10	1,1	1,1	96,4
	OTROS	8	,9	,9	97,2
	GDM Y OTROS	1	,1	,1	97,3
	GDM Y aborto	10	1,1	1,1	98,4
	desconocido	11	1,2	1,2	99,6
	HTA y aborto	4	,4	,4	100,0
	Total	932	100,0	100,0	

EDUCATIONAL_STATUS

		Frecuencia	Porcentaje	Porcentaje válido	Porcentaje acumulado
Válidos	SIN ESTUDIOS	1	,1	,1	,1
	PRIMARIOS	58	6,2	6,2	6,3
	BACHILLERATO	241	25,9	25,9	32,2
	FORMACION PROFESIONAL	145	15,6	15,6	47,8
	UNIVERSITARIO	476	51,1	51,1	98,9
	DESCONOCIDO	10	1,1	1,1	100,0
	Total	931	99,9	100,0	
Perdidos	Sistema	1	,1		
Total		932	100,0		

EMPLOYMENT

		Frecuencia	Porcentaje	Porcentaje válido	Porcentaje acumulado
Válidos	ESTUDIANTE	19	2,0	2,0	2,0
	AMA DE CASA	17	1,8	1,8	3,9
	FUERA DE CASA	743	79,7	79,7	83,6
	EN PARO	140	15,0	15,0	98,6
	5	2	,2	,2	98,8
	DESCONOCIDO	11	1,2	1,2	100,0
	Total	932	100,0	100,0	

NºPREGNANCY

		Frecuencia	Porcentaje	Porcentaje válido	Porcentaje acumulado
Válidos	1	394	42,3	42,6	42,6
	2	276	29,6	29,8	72,4
	3	171	18,3	18,5	90,9
	4	52	5,6	5,6	96,5
	5	23	2,5	2,5	99,0
	6	7	,8	,8	99,8
	7	2	,2	,2	100,0
	Total	925	99,2	100,0	
Perdidos	Sistema	7	,8		
Total		932	100,0		

SMOKE_SITUATION

		Frecuencia	Porcentaje	Porcentaje válido	Porcentaje acumulado
Válidos	nunca	531	57,0	57,0	57,0
	hasta hace 6 meses	205	22,0	22,0	79,0
	hasta embarazo	116	12,4	12,4	91,4
	fumo actualmente	77	8,3	8,3	99,7
	desconoce	3	,3	,3	100,0
	Total	932	100,0	100,0	

Estadísticos descriptivos

	N	Media	Desv. típ.
AGE	932	32,41	5,173
GESTATIONAL_AGE_AT_ENTRY	922	12,05	,535
kg	918	59,5909	9,74866
KG	924	61,3706	9,95044
COMPUTE GananciaPesoSG12=PESO_SG12 - Pesopregestacional	910	1,8183	3,36202
COMPUTE BMIPREGESTACIONAL=Pesopregestacional / (TALLAmetros * TALLAmetros)	915	22,4678	3,45147
COMPUTE BMISG12=PESO_SG12/ (TALLAmetros * TALLAmetros)	770	23,3969	3,72425

SBP_12GW	918	108,20	10,153
DBP_12GW	918	66,70	8,087
MG/DL	768	80,25	6,142
HbA1cSG12	55	5,115	,2422
CHOLESTROL_12GW	459	169,34	31,976
TRIGLICÉRIDOSSG12	450	78,75	34,367
TSHSG12	806	2,1298	2,85730
T4LSG12	777	8,7892	1,61639
COMPUTE SG12_MEDDIET_SCORE= SG12_ACEITEOLIVAMEDDIET + SG12_MLACEITEDIA_MEDDIET + SG12_VERDURAS_MEDDIET + SG12_FRUTAS_MEDDIET + SG12_CARNEPROCESADA_MEDDIET + SG12_MANTEQUILLA_MEDDIET + SG12_BEBIDASAZUCARADAS_MEDDIET + SG12_ALCOHOL_MEDDIET + SG12_LEGUMBRES_ME	744	4,3374	1,66523
PREG_NUTRITIONSCORE	793	,3884	3,05223
N válido (según lista)	43		

GRUPO MUNDO REAL VS CONTROL ES SUPERIOR

RR 0.82 (0.72-0.92) P <0.001

Estimación de riesgo

		Intervalo de confianza al 95%	
	Valor	Inferior	Superior
Razón de las ventajas para DIABETESGESTACIONAL (SI / NO)	1,673	1,252	2,235
Para la cohorte GRUPO_ALEATORIZADO = CONTROL	1,374	1,161	1,627

Para la cohorte GRUPO_ALEATORIZADO = INTERVENCION SIN ACEITE NI PISTACHOS	**,821**	**,727**	**,928**
N de casos válidos	1275		

Pruebas de chi-cuadrado

	Valor	gl	Sig. asintótica (bilateral)	Sig. exacta (bilateral)	Sig. exacta (unilateral)
Chi-cuadrado de Pearson	12,266[a]	1	,000		
Corrección por continuidad[b]	11,737	1	,001		
Razón de verosimilitudes	11,928	1	,001		
Estadístico exacto de Fisher				**,001**	,000
Asociación lineal por lineal	12,256	1	,000		
N de casos válidos	1275				

a. 0 casillas (,0%) tienen una frecuencia esperada inferior a 5. La frecuencia mínima esperada es 80,06.

b. Calculado sólo para una tabla de 2x2.

MUNDO REAL VS GRUPO INTERVENCIÓN NO ES DIFERENTE AUNQUE MEJOR PERO SIN SS

RR 0.96 (0.85-1.07) P =0.468

Estimación de riesgo

	Valor	Intervalo de confianza al 95%	
		Inferior	Superior
Razón de las ventajas para DIABETESGESTACIONAL (SI / NO)	1,125	,823	1,538
Para la cohorte GRUPO_ALEATORIZADO = INTERVENCION ACEITE OLIVA	1,079	,884	1,319
Para la cohorte GRUPO_ALEATORIZADO = INTERVENCION SIN ACEITE NI PISTACHOS	**,960**	**,857**	**1,074**
N de casos válidos	1269		

Pruebas de chi-cuadrado

	Valor	gl	Sig. asintótica (bilateral)	Sig. exacta (bilateral)	Sig. exacta (unilateral)
Chi-cuadrado de Pearson	,545[a]	1	,460		
Corrección por continuidad[b]	,432	1	,511		
Razón de verosimilitudes	,541	1	,462		
Estadístico exacto de Fisher				**,468**	,254
Asociación lineal por lineal	,545	1	,460		
N de casos válidos	1269				

a. 0 casillas (,0%) tienen una frecuencia esperada inferior a 5. La frecuencia mínima esperada es 69,43.

b. Calculado sólo para una tabla de 2x2.

Estadísticos de grupo

	GDM_DIAGNOSIS	N	Media	Desviación típ.	Error típ. de la media
SOG 75 G	SI	130	92,35	8,636	,757
	NO	802	82,90	4,883	,172
BG_60min_24GW	SI	76	164,07	33,547	3,848
	NO	798	118,33	26,439	,936
BG120min_24GW	SI	76	137,57	31,588	3,623
	NO	793	101,34	19,763	,702
SOG 75 G	SI	126	5,016	,3066	,0273
	NO	790	4,865	,2659	,0095
COLESTEROL_SG24	SI	116	238,44	43,062	3,998
	NO	700	243,12	41,571	1,571
TRIGLICÉRIDOS_SG24	SI	100	166,96	56,602	5,660
	NO	606	155,12	52,152	2,119
INSULINA_SG24	SI	71	10,015	6,5471	,7770
	NO	469	7,927	5,2783	,2437
HOMAIR_SG24	SI	69	2,337	1,5223	,1833
	NO	447	1,908	2,7451	,1298
TSH_SG24	SI	115	1,8690	1,33069	,12409
	NO	674	2,0031	1,02132	,03934
T4L_SG24	SI	113	6,9681	1,29481	,12181
	NO	670	6,9682	1,14747	,04433
KILOGRAMOS	SI	124	66,3057	10,33743	,92833
	NO	773	66,5423	9,96940	,35857
SBP_24GW	SI	121	106,43	11,036	1,003
	NO	755	104,84	10,848	,395
DBP_24GW	SI	121	64,75	9,253	,841
	NO	755	62,91	8,083	,294
KILOGRAMOS	SI	116	70,2517	11,30074	1,04925
	NO	594	71,3641	9,91473	,40681
SBP_36GW	SI	119	113,70	10,822	,992
	NO	698	114,50	13,528	,512
DBP_36GW	SI	119	70,22	9,912	,909
	NO	697	71,34	9,512	,360

Tabla de contingencia INSULINTREATEDGDM * GDM_DIAGNOSIS

Recuento

		GDM_DIAGNOSIS		
		SI	NO	Total
INSULINTREATEDGDM	1,00	30	0	30
	2,00	102	800	902
Total		130	802	932

Tabla de contingencia GDMTREATMENT * GDM_DIAGNOSIS

Recuento

		GDM_DIAGNOSIS		
		SI	NO	Total
GDMTREATMENT	DIETA	100	0	100
	INSULINA BASAL	18	0	18
	INSULINA BOLO INCOMPLETA	5	0	5
	INSULINA BASAL+BOLO INCOMPLETA	7	0	7
Total		130	0	130

Tabla de contingencia IWG_VS_ADEQUATE * GDM_DIAGNOSIS

Recuento

		GDM_DIAGNOSIS		
		SI	NO	Total
IWG_VS_ADEQUATE	,00	35	135	170
	1,00	61	296	357
Total		96	431	527

Tabla de contingencia EWG_VS_ADEQUATE * GDM_DIAGNOSIS

Recuento

		GDM_DIAGNOSIS		
		SI	NO	Total
EWG_VS_ADEQUATE	1,00	61	296	357
	2,00	34	371	405
Total		95	667	762

Prueba de muestras independientes

		Prueba de Levene para la igualdad de varianzas		Prueba T para la igualdad de medias						
									95% Intervalo de confianza para la diferencia	
		F	Sig.	t	gl	Sig. (bilateral)	Diferencia de medias	Error típ. de la diferencia	Inferior	Superior
SOG 75 G	Se han asumido varianzas iguales	38,583	,000	17,993	930	,000	9,454	,525	8,423	10,485
	No se han asumido varianzas iguales			12,169	142,652	,000	9,454	,777	7,918	10,989
BG_60min_24GW	Se han asumido varianzas iguales	10,519	,001	14,046	872	,000	45,736	3,256	39,345	52,127
	No se han asumido varianzas iguales			11,549	84,108	,000	45,736	3,960	37,861	53,612
BG120min_24GW	Se han asumido varianzas iguales	44,151	,000	14,332	867	,000	36,225	2,528	31,264	41,180
	No se han asumido varianzas iguales			9,815	80,722	,000	36,225	3,691	28,882	43,569
SOG 75 G	Se han asumido varianzas iguales	3,398	,066	5,778	914	,000	,1507	,0261	,0995	,2019
	No se han asumido varianzas iguales			5,213	156,443	,000	,1507	,0289	,0936	,2078
COLESTEROL_SG24	Se han asumido varianzas iguales	,004	,952	-1,117	814	,264	-4,679	4,189	-12,901	3,543
	No se han asumido varianzas iguales			-1,089	152,664	,278	-4,679	4,296	-13,166	3,808
TRIGLICÉRIDOS_SG24	Se han asumido varianzas iguales	1,958	,162	2,077	704	,038	11,835	5,699	,646	23,025
	No se han asumido varianzas iguales			1,958	128,269	,052	11,835	6,044	-,123	23,794
INSULINA_SG24	Se han asumido varianzas iguales	8,974	,003	3,003	538	,003	2,0882	,6953	,7223	3,454[illegible]

	No se han asumido varianzas iguales			2,564	84,331	,012	2,0882	,8143	,4689	3,707[illegible]
HOMAIR_SG24	Se han asumido varianzas iguales	,294	,588	1,269	514	,205	,4294	,3384	-,2354	1,094[illegible]
	No se han asumido varianzas iguales			1,912	147,715	,058	,4294	,2246	-,0144	,873[illegible]
TSH_SG24	Se han asumido varianzas iguales	,271	,603	-1,241	787	,215	-,13419	,10812	-,34644	,0780[illegible]
	No se han asumido varianzas iguales			-1,031	137,832	,304	-,13419	,13017	-,39159	,1232[illegible]
T4L_SG24	Se han asumido varianzas iguales	2,762	,097	-,001	781	,999	-,00008	,11896	-,23360	,2334[illegible]
	No se han asumido varianzas iguales			-,001	143,215	,999	-,00008	,12962	-,25630	,2561[illegible]
KILOGRAMOS	Se han asumido varianzas iguales	,241	,624	-,244	895	,807	-,23665	,96939	-2,13919	1,6658[illegible]
	No se han asumido varianzas iguales			-,238	161,866	,812	-,23665	,99517	-2,20185	1,7285[illegible]
SBP_24GW	Se han asumido varianzas iguales	,053	,819	1,494	874	,135	1,591	1,065	-,499	3,68[illegible]
	No se han asumido varianzas iguales			1,476	159,435	,142	1,591	1,078	-,538	3,72[illegible]
DBP_24GW	Se han asumido varianzas iguales	2,743	,098	2,278	874	,023	1,841	,808	,254	3,42[illegible]
	No se han asumido varianzas iguales			2,066	150,789	,041	1,841	,891	,080	3,60[illegible]
KILOGRAMOS	Se han asumido varianzas iguales	,293	,589	-1,079	708	,281	-1,11238	1,03060	-3,13578	,9110[illegible]
	No se han asumido varianzas iguales			-,988	151,508	,324	-1,11238	1,12535	-3,33579	1,1110[illegible]
SBP_36GW	Se han asumido varianzas iguales	5,557	,019	-,618	815	,537	-,807	1,306	-3,371	1,75[illegible]
	No se han asumido varianzas iguales			-,723	187,006	,471	-,807	1,116	-3,009	1,39[illegible]

DBP_36GW	Se han asumido varianzas iguales	,193	,661	-1,178	814	,239	-1,119	,949	-2,982	,74[illegible]
	No se han asumido varianzas iguales			-1,145	157,364	,254	-1,119	,977	-3,049	,81[illegible]

Estadísticos de grupo

	GDM_DIAGNOSIS	N	Media	Desviación típ.	Error típ. de la media
COMPUTE GananciaPesoSG12=PESO _SG12 - Pesopregestacional	SI	130	2,1285	4,70636	,41277
	NO	780	1,7666	3,08266	,11038
COMPUTE GananciaPesoSG24=PESO _SG24 - Pesopregestacional	SI	124	7,1258	4,90861	,44081
	NO	759	7,0208	4,27066	,15502
COMPUTE GananciaPesoSG38=PESO _SG36 - Pesopregestacional	SI	116	10,8828	6,46403	,60017
	NO	582	12,2990	5,42342	,22481
NUTRITION_SCORE_24G W	SI	106	2,7642	3,46012	,33608
	NO	716	2,3521	3,49342	,13056
NUTRITION_SCORE_36G W	SI	67	7,6119	3,20962	,39212
	NO	367	3,1580	3,45416	,18031
COMPUTE	SI	68	5,4412	1,57766	,19132

SG24_MEDDIET_SCORE= SG24_ACEITEOLIVAMEDDIET + SG24_MLACEITEDIA_MEDDIET + SG24_VERDURAS_MEDDIET + SG24_FRUTAS_MEDDIET + SG24_CARNEPROCESADA_MEDDIET + SG24_MANTEQUILLA_MEDDIET + SG24_BEBIDASAZUCARADAS_MEDDIET + SG24_ALCOHOL_MEDDIET + SG24_LEGUMBRES_ME	NO	491	5,2301	1,77108	,07993
COMPUTE	SI	37	7,6486	2,22631	,36600

SG36_MEDDIET_SCORE= SG36_ACEITEOLIVAMEDDIET + SG36_MLACEITEDIA_MEDDIET + SG36_VERDURAS_MEDDIET + SG36_FRUTAS_MEDDIET + SG36_CARNEPROCESADA_MEDDIET + SG36_MANTEQUILLA_MEDDIET + SG36_BEBIDASAZUCARADAS_MEDDIET + SG36_ALCOHOL_MEDDIET + SG36_LEGUMBRES_ME	NO	212	5,4104	1,78633	,12269

Prueba de muestras independientes

		Prueba de Levene para la igualdad de varianzas		Prueba T para la igualdad de medias						
									95% Intervalo de confianza pa[illegible] la diferencia	
		F	Sig.	t	gl	Sig. (bilateral)	Diferencia de medias	Error típ. de la diferencia	Inferior	Superior
COMPUTE GananciaPesoSG12=PESO_SG12 - Pesopregestacional	Se han asumido varianzas iguales	1,001	,317	1,136	908	,256	,36186	,31844	-,26311	,986[illegible]
	No se han asumido varianzas iguales			,847	147,982	,398	,36186	,42728	-,48249	1,206[illegible]
COMPUTE GananciaPesoSG24=PESO_SG24 - Pesopregestacional	Se han asumido varianzas iguales	,008	,927	,248	881	,804	,10506	,42283	-,72481	,934[illegible]
	No se han asumido varianzas iguales			,225	154,919	,822	,10506	,46727	-,81798	1,028[illegible]

COMPUTE GananciaPesoSG38=PESO_SG36 - Pesopregestacional	Se han asumido varianzas iguales	,245	,621	-2,483	696	,013	-1,41626	,57029	-2,53596	-,296
	No se han asumido varianzas iguales			-2,210	148,954	,029	-1,41626	,64089	-2,68268	-,149
NUTRITION_SCORE_24GW	Se han asumido varianzas iguales	,011	,917	1,135	820	,257	,41206	,36312	-,30070	1,124
	No se han asumido varianzas iguales			1,143	138,618	,255	,41206	,36054	-,30082	1,124
NUTRITION_SCORE_36GW	Se han asumido varianzas iguales	,901	,343	9,808	432	,000	4,45390	,45409	3,56141	5,346
	No se han asumido varianzas iguales			10,320	96,086	,000	4,45390	,43159	3,59722	5,310
COMPUTE SG24_MEDDIET_SCORE= SG24_ACEITEOLIVAMEDDIET + SG24_MLACEITEDIA_MEDDIET + SG24_VERDURAS_MEDDIET + SG24_FRUTAS_MEDDIET + SG24_CARNEPROCESADA_MEDDIET + SG24_MANTEQUILLA_MEDDIET + SG24_BEBIDASAZUCARADAS_MEDDIET + SG24_ALCOHOL_MEDDIET + SG24_LEGUMBRES_ME	Se han asumido varianzas iguales	1,111	,292	,933	557	,351	,21103	,22630	-,23347	,655
	No se han asumido varianzas iguales			1,018	92,045	,311	,21103	,20734	-,20077	,622
COMPUTE SG36_MEDDIET_SCORE=	Se han asumido varianzas iguales	6,115	,014	6,765	247	,000	2,23827	,33085	1,58662	2,889

SG36_ACEITEOLIVAMEDDIET + SG36_MLACEITEDIA_MEDDIET + SG36_VERDURAS_MEDDIET + SG36_FRUTAS_MEDDIET + SG36_CARNEPROCESADA_MEDDIET + SG36_MANTEQUILLA_MEDDIET + SG36_BEBIDASAZUCARADAS_MEDDIET + SG36_ALCOHOL_MEDDIET + SG36_LEGUMBRES_ME	No se han asumido varianzas iguales			5,798	44,449	,000	2,23827	,38602	1,46052	3,016

Estadísticos de grupo

	GDM_DIAGNOSIS	N	Media	Desviación típ.	Error típ. de la media
GW_AT_DELIVERY	SI	123	39,4614	1,68391	,15183
	NO	734	39,5799	1,44956	,05350
Peso RN en gramos	SI	114	3126,3333	465,05159	43,55607
	NO	697	3273,0344	468,27065	17,73702
PERCENTIL_BW	SI	114	41,78	28,274	2,648
	NO	691	49,64	27,825	1,059
LENGTH_cm	SI	67	49,0597	1,93360	,23623
	NO	486	49,4105	2,13119	,09667
PERCENTIL_TALLA	SI	67	38,72	26,838	3,279
	NO	486	42,71	27,432	1,244
PHCORD_BLOOD	SI	107	7,2668	,08470	,00819
	NO	672	7,2779	,13352	,00515

APGAR_1MIN	SI	107	8,79	,762	,074
	NO	695	8,78	,958	,036
APGAR_5MIN	SI	107	9,90	,305	,029
	NO	697	9,81	,658	,025

Prueba de muestras independientes

		Prueba de Levene para la igualdad de varianzas		Prueba T para la igualdad de medias						
									95% Intervalo de confianza para la diferencia	
		F	Sig.	t	gl	Sig. (bilateral)	Diferencia de medias	Error típ. de la diferencia	Inferior	Superior
GW_AT_DELIVERY	Se han asumido varianzas iguales	1,678	,196	-,819	855	,413	-,11854	,14471	-,40256	,16549
	No se han asumido varianzas iguales			-,736	153,786	,463	-,11854	,16098	-,43656	,19949
Peso RN en gramos	Se han asumido varianzas iguales	,098	,754	-3,104	809	,002	-146,70110	47,26314	-239,47395	-53,92825
	No se han asumido varianzas iguales			-3,119	152,902	,002	-146,70110	47,02907	-239,61174	-53,79046
PERCENTIL_BW	Se han asumido varianzas iguales	,724	,395	-2,787	803	,005	-7,856	2,819	-13,390	-2,322
	No se han asumido varianzas iguales			-2,755	151,364	,007	-7,856	2,852	-13,491	-2,222
LENGTH_cm	Se han asumido varianzas iguales	,057	,811	-1,277	551	,202	-,35079	,27478	-,89053	,18895
	No se han asumido varianzas iguales			-1,374	89,616	,173	-,35079	,25524	-,85791	,15632
PERCENTIL_TALLA	Se han asumido varianzas iguales	,026	,872	-1,120	551	,263	-3,993	3,566	-10,998	3,011
	No se han asumido varianzas iguales			-1,139	86,138	,258	-3,993	3,507	-10,965	2,978
PHCORD_BLOOD	Se han asumido varianzas iguales	,000	,983	-,833	777	,405	-,01109	,01332	-,03724	,01505

	No se han asumido varianzas iguales			-1,147	201,491	,253	-,01109	,00967	-,03017	,00798
APGAR_1MIN	Se han asumido varianzas iguales	1,383	,240	,194	800	,846	,019	,097	-,172	,209
	No se han asumido varianzas iguales			,230	162,473	,819	,019	,082	-,143	,181
APGAR_5MIN	Se han asumido varianzas iguales	6,040	,014	1,293	802	,196	,084	,065	-,043	,211
	No se han asumido varianzas iguales			2,167	289,209	,031	,084	,039	,008	,160

Tabla de contingencia

			GDM_DIAGNOSIS		Total
			SI	NO	
IWG_VS_ADEQUATE	,00	Recuento	35	135	170
		% dentro de GDM_DIAGNOSIS	36,5%	31,3%	32,3%
	1,00	Recuento	61	296	357
		% dentro de GDM_DIAGNOSIS	63,5%	68,7%	67,7%
Total		Recuento	96	431	527
		% dentro de GDM_DIAGNOSIS	100,0%	100,0%	100,0%

Pruebas de chi-cuadrado

	Valor	gl	Sig. asintótica (bilateral)	Sig. exacta (bilateral)	Sig. exacta (unilateral)
Chi-cuadrado de Pearson	,948[a]	1	,330		
Corrección por continuidad[b]	,727	1	,394		
Razón de verosimilitudes	,932	1	,334		
Estadístico exacto de Fisher				,336	,196

Asociación lineal por lineal	,946	1	,331		
N de casos válidos	527				

a. 0 casillas (,0%) tienen una frecuencia esperada inferior a 5. La frecuencia mínima esperada es 30,97.

b. Calculado sólo para una tabla de 2x2.

Estimación de riesgo

	Valor	Intervalo de confianza al 95%	
		Inferior	Superior
Razón de las ventajas para IWG_VS_ADEQUATE (,00 / 1,00)	1,258	,792	1,998
Para la cohorte GDM_DIAGNOSIS = SI	1,205	,830	1,750
Para la cohorte GDM_DIAGNOSIS = NO	,958	,875	1,048
N de casos válidos	527		

Tabla de contingencia

			GDM_DIAGNOSIS		Total
			SI	NO	
EWG_VS_ADEQUATE	1,00	Recuento	61	296	357
		% dentro de GDM_DIAGNOSIS	64,2%	44,4%	46,9%
	2,00	Recuento	34	371	405
		% dentro de GDM_DIAGNOSIS	35,8%	55,6%	53,1%
Total		Recuento	95	667	762
		% dentro de GDM_DIAGNOSIS	100,0%	100,0%	100,0%

Pruebas de chi-cuadrado

	Valor	gl	Sig. asintótica (bilateral)	Sig. exacta (bilateral)	Sig. exacta (unilateral)
Chi-cuadrado de Pearson	13,135[a]	1	,000		
Corrección por continuidad[b]	12,351	1	,000		
Razón de verosimilitudes	13,206	1	,000		
Estadístico exacto de Fisher				,000	,000
Asociación lineal por lineal	13,118	1	,000		
N de casos válidos	762				

a. 0 casillas (,0%) tienen una frecuencia esperada inferior a 5. La frecuencia mínima esperada es 44,51.

b. Calculado sólo para una tabla de 2x2.

Estimación de riesgo

	Valor	Intervalo de confianza al 95%	
		Inferior	Superior
Razón de las ventajas para EWG_VS_ADEQUATE (1,00 / 2,00)	2,249	1,439	3,514
Para la cohorte GDM_DIAGNOSIS = SI	2,035	1,372	3,020
Para la cohorte GDM_DIAGNOSIS = NO	,905	,856	,957
N de casos válidos	762		

Tabla de contingencia

			GDM_DIAGNOSIS		Total
			SI	NO	
PREGNACY_INDUCED_HYPERTENSION	GESTACIONAL	Recuento	3	15	18
		% dentro de GDM_DIAGNOSIS	2,3%	1,9%	1,9%

	PREGESTACIONAL	Recuento	127	787	914
		% dentro de GDM_DIAGNOSIS	97,7%	98,1%	98,1%
Total		Recuento	130	802	932
		% dentro de GDM_DIAGNOSIS	100,0%	100,0%	100,0%

Pruebas de chi-cuadrado

	Valor	gl	Sig. asintótica (bilateral)	Sig. exacta (bilateral)	Sig. exacta (unilateral)
Chi-cuadrado de Pearson	,113[a]	1	,737		
Corrección por continuidad[b]	,000	1	1,000		
Razón de verosimilitudes	,107	1	,743		
Estadístico exacto de Fisher				,729	,470
Asociación lineal por lineal	,113	1	,737		
N de casos válidos	932				

a. 1 casillas (25,0%) tienen una frecuencia esperada inferior a 5. La frecuencia mínima esperada es 2,51.

b. Calculado sólo para una tabla de 2x2.

Estimación de riesgo

	Valor	Intervalo de confianza al 95%	
		Inferior	Superior
Razón de las ventajas para PREGNACY_INDUCED_HYPERTENSION (GESTACIONAL / PREGESTACIONAL)	1,239	,354	4,342
Para la cohorte GDM_DIAGNOSIS = SI	1,199	,422	3,412
Para la cohorte GDM_DIAGNOSIS = NO	,968	,786	1,192

Estimación de riesgo

	Valor	Intervalo de confianza al 95%	
		Inferior	Superior
Razón de las ventajas para PREGNACY_INDUCED_HYPERTENSION (GESTACIONAL / PREGESTACIONAL)	1,239	,354	4,342
Para la cohorte GDM_DIAGNOSIS = SI	1,199	,422	3,412
Para la cohorte GDM_DIAGNOSIS = NO	,968	,786	1,192
N de casos válidos	932		

Tabla de contingencia

			GDM_DIAGNOSIS		Total
			SI	NO	
PRECLAMPSIA_ECLAMPSIA	SI	Recuento	1	9	10
		% dentro de GDM_DIAGNOSIS	,8%	1,1%	1,1%
	NO	Recuento	129	793	922
		% dentro de GDM_DIAGNOSIS	99,2%	98,9%	98,9%
Total		Recuento	130	802	932
		% dentro de GDM_DIAGNOSIS	100,0%	100,0%	100,0%

Pruebas de chi-cuadrado

	Valor	gl	Sig. asintótica (bilateral)	Sig. exacta (bilateral)	Sig. exacta (unilateral)

Chi-cuadrado de Pearson	,131[a]	1	,717		
Corrección por continuidad[b]	,000	1	1,000		
Razón de verosimilitudes	,143	1	,705		
Estadístico exacto de Fisher				1,000	,583
Asociación lineal por lineal	,131	1	,717		
N de casos válidos	932				

a. 1 casillas (25,0%) tienen una frecuencia esperada inferior a 5. La frecuencia mínima esperada es 1,39.

b. Calculado sólo para una tabla de 2x2.

Estimación de riesgo

	Valor	Intervalo de confianza al 95%	
		Inferior	Superior
Razón de las ventajas para PRECLAMPSIA_ECLAMPSIA (SI / NO)	,683	,086	5,436
Para la cohorte GDM_DIAGNOSIS = SI	,715	,111	4,620
Para la cohorte GDM_DIAGNOSIS = NO	1,046	,850	1,289
N de casos válidos	932		

Tabla de contingencia

			GDM_DIAGNOSIS		Total
			SI	NO	
PROTEINURIA	SI	Recuento	2	4	6
		% dentro de GDM_DIAGNOSIS	1,5%	,5%	,6%
	NO	Recuento	128	798	926
		% dentro de GDM_DIAGNOSIS	98,5%	99,5%	99,4%
Total		Recuento	130	802	932

Tabla de contingencia

			GDM_DIAGNOSIS		Total
			SI	NO	
PROTEINURIA	SI	Recuento	2	4	6
		% dentro de GDM_DIAGNOSIS	1,5%	,5%	,6%
	NO	Recuento	128	798	926
		% dentro de GDM_DIAGNOSIS	98,5%	99,5%	99,4%
Total		Recuento	130	802	932
		% dentro de GDM_DIAGNOSIS	100,0%	100,0%	100,0%

Pruebas de chi-cuadrado

	Valor	gl	Sig. asintótica (bilateral)	Sig. exacta (bilateral)	Sig. exacta (unilateral)
Chi-cuadrado de Pearson	1,891[a]	1	,169		
Corrección por continuidad[b]	,614	1	,433		
Razón de verosimilitudes	1,455	1	,228		
Estadístico exacto de Fisher				,199	,199
Asociación lineal por lineal	1,889	1	,169		
N de casos válidos	932				

a. 1 casillas (25,0%) tienen una frecuencia esperada inferior a 5. La frecuencia mínima esperada es ,84.

b. Calculado sólo para una tabla de 2x2.

Estimación de riesgo

	Valor	Intervalo de confianza al 95%	
		Inferior	Superior
Razón de las ventajas para PROTEINURIA (SI / NO)	3,117	,565	17,194

Para la cohorte GDM_DIAGNOSIS = SI	2,411	,769	7,562
Para la cohorte GDM_DIAGNOSIS = NO	,774	,439	1,363
N de casos válidos	932		

Tabla de contingencia

			GDM_DIAGNOSIS		
			SI	NO	Total
BACTERIURIA	SI	Recuento	35	120	155
		% dentro de GDM_DIAGNOSIS	26,9%	15,0%	16,6%
	NO	Recuento	95	682	777
		% dentro de GDM_DIAGNOSIS	73,1%	85,0%	83,4%
Total		Recuento	130	802	932
		% dentro de GDM_DIAGNOSIS	100,0%	100,0%	100,0%

Pruebas de chi-cuadrado

	Valor	gl	Sig. asintótica (bilateral)	Sig. exacta (bilateral)	Sig. exacta (unilateral)
Chi-cuadrado de Pearson	11,542[a]	1	,001		
Corrección por continuidad[b]	10,695	1	,001		
Razón de verosimilitudes	10,341	1	,001		
Estadístico exacto de Fisher				,001	,001
Asociación lineal por lineal	11,530	1	,001		
N de casos válidos	932				

a. 0 casillas (,0%) tienen una frecuencia esperada inferior a 5. La frecuencia mínima esperada es 21,62.

b. Calculado sólo para una tabla de 2x2.

Estimación de riesgo

	Valor	Intervalo de confianza al 95%	
		Inferior	Superior
Razón de las ventajas para BACTERIURIA (SI / NO)	2,094	1,357	3,230
Para la cohorte GDM_DIAGNOSIS = SI	1,847	1,305	2,613
Para la cohorte GDM_DIAGNOSIS = NO	,882	,807	,964
N de casos válidos	932		

Tabla de contingencia

			GDM_DIAGNOSIS		Total
			SI	NO	
UTI	SI	Recuento	7	31	38
		% dentro de GDM_DIAGNOSIS	5,4%	3,9%	4,1%
	NO	Recuento	123	771	894
		% dentro de GDM_DIAGNOSIS	94,6%	96,1%	95,9%
Total		Recuento	130	802	932
		% dentro de GDM_DIAGNOSIS	100,0%	100,0%	100,0%

Pruebas de chi-cuadrado

	Valor	gl	Sig. asintótica (bilateral)	Sig. exacta (bilateral)	Sig. exacta (unilateral)
Chi-cuadrado de Pearson	,660[a]	1	,416		
Corrección por continuidad[b]	,329	1	,566		
Razón de verosimilitudes	,612	1	,434		

Estadístico exacto de Fisher				,470	,271
Asociación lineal por lineal	,660	1	,417		
N de casos válidos	932				

a. 0 casillas (,0%) tienen una frecuencia esperada inferior a 5. La frecuencia mínima esperada es 5,30.

b. Calculado sólo para una tabla de 2x2.

Estimación de riesgo

	Valor	Intervalo de confianza al 95%	
		Inferior	Superior
Razón de las ventajas para UTI (SI / NO)	1,415	,610	3,285
Para la cohorte GDM_DIAGNOSIS = SI	1,339	,672	2,667
Para la cohorte GDM_DIAGNOSIS = NO	,946	,811	1,103
N de casos válidos	932		

Tabla de contingencia

			GDM_DIAGNOSIS		Total
			SI	NO	
DELIVERY	EUTOCICO	Recuento	76	503	579
		% dentro de GDM_DIAGNOSIS	58,5%	62,7%	62,1%
	INSTRUMENTAL	Recuento	30	129	159
		% dentro de GDM_DIAGNOSIS	23,1%	16,1%	17,1%
	CESAREA	Recuento	24	170	194
		% dentro de GDM_DIAGNOSIS	18,5%	21,2%	20,8%
Total		Recuento	130	802	932
		% dentro de GDM_DIAGNOSIS	100,0%	100,0%	100,0%

Pruebas de chi-cuadrado

	Valor	gl	Sig. asintótica (bilateral)
Chi-cuadrado de Pearson	3,934[a]	2	,140
Razón de verosimilitudes	3,685	2	,158
Asociación lineal por lineal	,039	1	,843
N de casos válidos	932		

a. 0 casillas (,0%) tienen una frecuencia esperada inferior a 5. La frecuencia mínima esperada es 22,18.

Tabla de contingencia

			GDM_DIAGNOSIS		Total
			SI	NO	
EMERGENCY_CS	C. ELECTIVA PRIMER PARTO NALGAS	Recuento	4	25	29
		% dentro de GDM_DIAGNOSIS	18,2%	15,3%	15,7%
	C. ELECTIVA PLACENT PREVIA	Recuento	1	7	8
		% dentro de GDM_DIAGNOSIS	4,5%	4,3%	4,3%
	C. ELECTIVA 2 CESAREAS PREVIAS	Recuento	0	6	6
		% dentro de GDM_DIAGNOSIS	,0%	3,7%	3,2%
	C. ELECTIVA MIOMA	Recuento	0	1	1
		% dentro de GDM_DIAGNOSIS	,0%	,6%	,5%
	C. ELECTIVA CRONOLOGICAMENTE PROLONGADA	Recuento	6	28	34
		% dentro de GDM_DIAGNOSIS	27,3%	17,2%	18,4%
	C. ELECTIVA INTERES	Recuento	6	39	45

	MATERNO	% dentro de GDM_DIAGNOSIS	27,3%	23,9%	24,3%
	C. ELECTIVA OTRAS	Recuento	2	35	37
		% dentro de GDM_DIAGNOSIS	9,1%	21,5%	20,0%
	C. URGENTE SUFRIMIENTO FETAL	Recuento	1	1	2
		% dentro de GDM_DIAGNOSIS	4,5%	,6%	1,1%
	C. URGENTE DESPROPORCION PELVICA	Recuento	0	1	1
		% dentro de GDM_DIAGNOSIS	,0%	,6%	,5%
	C. URGENTE ROTURA UTERINA	Recuento	2	19	21
		% dentro de GDM_DIAGNOSIS	9,1%	11,7%	11,4%
	C. URGENTE NO PROGRESION	Recuento	0	1	1
		% dentro de GDM_DIAGNOSIS	,0%	,6%	,5%
Total		Recuento	22	163	185
		% dentro de GDM_DIAGNOSIS	100,0%	100,0%	100,0%

Pruebas de chi-cuadrado

	Valor	gl	Sig. asintótica (bilateral)
Chi-cuadrado de Pearson	6,852[a]	10	,739
Razón de verosimilitudes	7,095	10	,716
Asociación lineal por lineal	,309	1	,579
N de casos válidos	185		

Pruebas de chi-cuadrado

	Valor	gl	Sig. asintótica (bilateral)
Chi-cuadrado de Pearson	6,852[a]	10	,739
Razón de verosimilitudes	7,095	10	,716
Asociación lineal por lineal	,309	1	,579
N de casos válidos	185		

a. 14 casillas (63,6%) tienen una frecuencia esperada inferior a 5. La frecuencia mínima esperada es ,12.

Estimación de riesgo

	Valor	Intervalo de confianza al 95%	
		Inferior	Superior
Razón de las ventajas para cesareasi_no (1,00 / 2,00)	,842	,524	1,353
Para la cohorte GDM_DIAGNOSIS = SI	,861	,569	1,303
Para la cohorte GDM_DIAGNOSIS = NO	1,023	,963	1,087
N de casos válidos	932		

Tabla de contingencia

			GDM_DIAGNOSIS		Total
			SI	NO	
INDUCTION_YesNon	SI	Recuento	78	490	568
		% dentro de GDM_DIAGNOSIS	60,0%	61,1%	60,9%
	NO	Recuento	52	312	364
		% dentro de GDM_DIAGNOSIS	40,0%	38,9%	39,1%

Total	Recuento	130	802	932
	% dentro de GDM_DIAGNOSIS	100,0%	100,0%	100,0%

Pruebas de chi-cuadrado

	Valor	gl	Sig. asintótica (bilateral)	Sig. exacta (bilateral)	Sig. exacta (unilateral)
Chi-cuadrado de Pearson	,057[a]	1	,812		
Corrección por continuidad[b]	,020	1	,888		
Razón de verosimilitudes	,056	1	,812		
Estadístico exacto de Fisher				,847	,442
Asociación lineal por lineal	,057	1	,812		
N de casos válidos	932				

a. 0 casillas (,0%) tienen una frecuencia esperada inferior a 5. La frecuencia mínima esperada es 50,77.

b. Calculado sólo para una tabla de 2x2.

Estimación de riesgo

	Valor	Intervalo de confianza al 95%	
		Inferior	Superior
Razón de las ventajas para INDUCTION_YesNon (SI / NO)	,955	,654	1,395
Para la cohorte GDM_DIAGNOSIS = SI	,961	,694	1,331
Para la cohorte GDM_DIAGNOSIS = NO	1,006	,954	1,062
N de casos válidos	932		

Tabla de contingencia

	GDM_DIAGNOSIS		Total
	SI	NO	

ANALGESIA_SINO	SI	Recuento	106	661	767
		% dentro de GDM_DIAGNOSIS	81,5%	82,4%	82,3%
	NO	Recuento	24	141	165
		% dentro de GDM_DIAGNOSIS	18,5%	17,6%	17,7%
Total		Recuento	130	802	932
		% dentro de GDM_DIAGNOSIS	100,0%	100,0%	100,0%

Pruebas de chi-cuadrado

	Valor	gl	Sig. asintótica (bilateral)	Sig. exacta (bilateral)	Sig. exacta (unilateral)
Chi-cuadrado de Pearson	,060[a]	1	,807		
Corrección por continuidad[b]	,014	1	,904		
Razón de verosimilitudes	,059	1	,808		
Estadístico exacto de Fisher				,805	,445
Asociación lineal por lineal	,059	1	,807		
N de casos válidos	932				

a. 0 casillas (,0%) tienen una frecuencia esperada inferior a 5. La frecuencia mínima esperada es 23,02.

b. Calculado sólo para una tabla de 2x2.

Estimación de riesgo

	Valor	Intervalo de confianza al 95%	
		Inferior	Superior
Razón de las ventajas para ANALGESIA_SINO (SI / NO)	,942	,584	1,521
Para la cohorte GDM_DIAGNOSIS = SI	,950	,631	1,432
Para la cohorte GDM_DIAGNOSIS = NO	1,008	,941	1,081

Estimación de riesgo

	Valor	Intervalo de confianza al 95% Inferior	Superior
Razón de las ventajas para ANALGESIA_SINO (SI / NO)	,942	,584	1,521
Para la cohorte GDM_DIAGNOSIS = SI	,950	,631	1,432
Para la cohorte GDM_DIAGNOSIS = NO	1,008	,941	1,081
N de casos válidos	932		

Tabla de contingencia

			GDM_DIAGNOSIS SI	NO	Total
EPISOTOMY_SINO	SI	Recuento	43	256	299
		% dentro de GDM_DIAGNOSIS	33,1%	31,9%	32,1%
	NO	Recuento	87	546	633
		% dentro de GDM_DIAGNOSIS	66,9%	68,1%	67,9%
Total		Recuento	130	802	932
		% dentro de GDM_DIAGNOSIS	100,0%	100,0%	100,0%

Pruebas de chi-cuadrado

	Valor	gl	Sig. asintótica (bilateral)	Sig. exacta (bilateral)	Sig. exacta (unilateral)
Chi-cuadrado de Pearson	,069[a]	1	,793		
Corrección por continuidad[b]	,026	1	,872		
Razón de verosimilitudes	,068	1	,794		

Estadístico exacto de Fisher				,840	,433
Asociación lineal por lineal	,069	1	,793		
N de casos válidos	932				

a. 0 casillas (,0%) tienen una frecuencia esperada inferior a 5. La frecuencia mínima esperada es 41,71.

b. Calculado sólo para una tabla de 2x2.

Estimación de riesgo

	Valor	Intervalo de confianza al 95%	
		Inferior	Superior
Razón de las ventajas para EPISOTOMY_SINO (SI / NO)	1,054	,711	1,564
Para la cohorte GDM_DIAGNOSIS = SI	1,046	,746	1,468
Para la cohorte GDM_DIAGNOSIS = NO	,993	,939	1,050
N de casos válidos	932		

Tabla de contingencia

			GDM_DIAGNOSIS		Total
			SI	NO	
PERINEAL_TRAUMA_Y_N	SI	Recuento	36	265	301
		% dentro de GDM_DIAGNOSIS	27,7%	33,0%	32,3%
	NO	Recuento	94	537	631
		% dentro de GDM_DIAGNOSIS	72,3%	67,0%	67,7%
Total		Recuento	130	802	932

Tabla de contingencia

			GDM_DIAGNOSIS		Total
			SI	NO	
PERINEAL_TRAUMA_Y_N	SI	Recuento	36	265	301
		% dentro de GDM_DIAGNOSIS	27,7%	33,0%	32,3%
	NO	Recuento	94	537	631
		% dentro de GDM_DIAGNOSIS	72,3%	67,0%	67,7%
Total		Recuento	130	802	932
		% dentro de GDM_DIAGNOSIS	100,0%	100,0%	100,0%

Pruebas de chi-cuadrado

	Valor	gl	Sig. asintótica (bilateral)	Sig. exacta (bilateral)	Sig. exacta (unilateral)
Chi-cuadrado de Pearson	1,464[a]	1	,226		
Corrección por continuidad[b]	1,230	1	,267		
Razón de verosimilitudes	1,498	1	,221		
Estadístico exacto de Fisher				,266	,133
Asociación lineal por lineal	1,463	1	,226		
N de casos válidos	932				

a. 0 casillas (,0%) tienen una frecuencia esperada inferior a 5. La frecuencia mínima esperada es 41,98.

b. Calculado sólo para una tabla de 2x2.

Estimación de riesgo

	Valor	Intervalo de confianza al 95%	
		Inferior	Superior

Razón de las ventajas para PERINEAL_TRAUMA_Y_N (SI / NO)	,776	,514	1,171
Para la cohorte GDM_DIAGNOSIS = SI	,803	,561	1,149
Para la cohorte GDM_DIAGNOSIS = NO	1,035	,981	1,091
N de casos válidos	932		

Tabla de contingencia

			GDM_DIAGNOSIS		
			SI	NO	Total
PREMATURITY_37	1,00	Recuento	7	46	53
		% dentro de GDM_DIAGNOSIS	5,4%	5,7%	5,7%
	2,00	Recuento	123	756	879
		% dentro de GDM_DIAGNOSIS	94,6%	94,3%	94,3%
Total		Recuento	130	802	932
		% dentro de GDM_DIAGNOSIS	100,0%	100,0%	100,0%

Pruebas de chi-cuadrado

	Valor	gl	Sig. asintótica (bilateral)	Sig. exacta (bilateral)	Sig. exacta (unilateral)
Chi-cuadrado de Pearson	,026[a]	1	,873		
Corrección por continuidad[b]	,000	1	1,000		
Razón de verosimilitudes	,026	1	,872		
Estadístico exacto de Fisher				1,000	,535
Asociación lineal por lineal	,026	1	,873		
N de casos válidos	932				

Pruebas de chi-cuadrado

	Valor	gl	Sig. asintótica (bilateral)	Sig. exacta (bilateral)	Sig. exacta (unilateral)
Chi-cuadrado de Pearson	,026[a]	1	,873		
Corrección por continuidad[b]	,000	1	1,000		
Razón de verosimilitudes	,026	1	,872		
Estadístico exacto de Fisher				1,000	,535
Asociación lineal por lineal	,026	1	,873		
N de casos válidos	932				

a. 0 casillas (,0%) tienen una frecuencia esperada inferior a 5. La frecuencia mínima esperada es 7,39.

b. Calculado sólo para una tabla de 2x2.

Estimación de riesgo

	Valor	Intervalo de confianza al 95%	
		Inferior	Superior
Razón de las ventajas para PREMATURITY_37 (1,00 / 2,00)	,935	,413	2,119
Para la cohorte GDM_DIAGNOSIS = SI	,944	,464	1,919
Para la cohorte GDM_DIAGNOSIS = NO	1,009	,906	1,125
N de casos válidos	932		

Tabla de contingencia

			GDM_DIAGNOSIS		Total
			SI	NO	
LGA_4000_4500	1,00	Recuento	1	31	32
		% dentro de GDM_DIAGNOSIS	,8%	3,9%	3,4%
	2,00	Recuento	129	771	900

	% dentro de GDM_DIAGNOSIS	99,2%	96,1%	96,6%
Total	Recuento	130	802	932
	% dentro de GDM_DIAGNOSIS	100,0%	100,0%	100,0%

Pruebas de chi-cuadrado

	Valor	gl	Sig. asintótica (bilateral)	Sig. exacta (bilateral)	Sig. exacta (unilateral)
Chi-cuadrado de Pearson	3,234[a]	1	,072		
Corrección por continuidad[b]	2,368	1	,124		
Razón de verosimilitudes	4,464	1	,035		
Estadístico exacto de Fisher				,113	,048
Asociación lineal por lineal	3,231	1	,072		
N de casos válidos	932				

a. 1 casillas (25,0%) tienen una frecuencia esperada inferior a 5. La frecuencia mínima esperada es 4,46.

b. Calculado sólo para una tabla de 2x2.

Estimación de riesgo

	Valor	Intervalo de confianza al 95%	
		Inferior	Superior
Razón de las ventajas para LGA_4000_4500 (1,00 / 2,00)	,193	,026	1,425
Para la cohorte GDM_DIAGNOSIS = SI	,218	,031	1,511
Para la cohorte GDM_DIAGNOSIS = NO	1,131	1,057	1,210
N de casos válidos	932		

Tabla de contingencia

			GDM_DIAGNOSIS		Total
			SI	NO	
SGA_2000_2500	1,00	Recuento	5	30	35
		% dentro de GDM_DIAGNOSIS	3,8%	3,7%	3,8%
	2,00	Recuento	125	772	897
		% dentro de GDM_DIAGNOSIS	96,2%	96,3%	96,2%
Total		Recuento	130	802	932
		% dentro de GDM_DIAGNOSIS	100,0%	100,0%	100,0%

Pruebas de chi-cuadrado

	Valor	gl	Sig. asintótica (bilateral)	Sig. exacta (bilateral)	Sig. exacta (unilateral)
Chi-cuadrado de Pearson	,003[a]	1	,953		
Corrección por continuidad[b]	,000	1	1,000		
Razón de verosimilitudes	,003	1	,953		
Estadístico exacto de Fisher				1,000	,553
Asociación lineal por lineal	,003	1	,953		
N de casos válidos	932				

a. 1 casillas (25,0%) tienen una frecuencia esperada inferior a 5. La frecuencia mínima esperada es 4,88.

b. Calculado sólo para una tabla de 2x2.

Estimación de riesgo

	Valor	Intervalo de confianza al 95%	
		Inferior	Superior

Razón de las ventajas para SGA_2000_2500 (1,00 / 2,00)	1,029	,392	2,703
Para la cohorte GDM_DIAGNOSIS = SI	1,025	,448	2,345
Para la cohorte GDM_DIAGNOSIS = NO	,996	,868	1,143
N de casos válidos	932		

Tabla de contingencia

			GDM_DIAGNOSIS		Total
			SI	NO	
pH7.1	1,00	Recuento	6	19	25
		% dentro de GDM_DIAGNOSIS	4,6%	2,4%	2,7%
	2,00	Recuento	124	783	907
		% dentro de GDM_DIAGNOSIS	95,4%	97,6%	97,3%
Total		Recuento	130	802	932
		% dentro de GDM_DIAGNOSIS	100,0%	100,0%	100,0%

Pruebas de chi-cuadrado

	Valor	gl	Sig. asintótica (bilateral)	Sig. exacta (bilateral)	Sig. exacta (unilateral)
Chi-cuadrado de Pearson	2,162[a]	1	,141		
Corrección por continuidad[b]	1,387	1	,239		
Razón de verosimilitudes	1,850	1	,174		
Estadístico exacto de Fisher				,144	,122
Asociación lineal por lineal	2,160	1	,142		

N de casos válidos	932					

a. 1 casillas (25,0%) tienen una frecuencia esperada inferior a 5. La frecuencia mínima esperada es 3,49.

b. Calculado sólo para una tabla de 2x2.

Estimación de riesgo

	Valor	Intervalo de confianza al 95%	
		Inferior	Superior
Razón de las ventajas para pH7.1 (1,00 / 2,00)	1,994	,781	5,090
Para la cohorte GDM_DIAGNOSIS = SI	1,755	,858	3,594
Para la cohorte GDM_DIAGNOSIS = NO	,880	,705	1,099
N de casos válidos	932		

Tabla de contingencia

			GDM_DIAGNOSIS		Total
			SI	NO	
Apgar_1min_5	1,00	Recuento	1	11	12
		% dentro de GDM_DIAGNOSIS	,8%	1,4%	1,3%
	2,00	Recuento	129	791	920
		% dentro de GDM_DIAGNOSIS	99,2%	98,6%	98,7%
Total		Recuento	130	802	932
		% dentro de GDM_DIAGNOSIS	100,0%	100,0%	100,0%

Pruebas de chi-cuadrado

	Valor	gl	Sig. asintótica (bilateral)	Sig. exacta (bilateral)	Sig. exacta (unilateral)
Chi-cuadrado de Pearson	,319[a]	1	,572		
Corrección por continuidad[b]	,021	1	,884		
Razón de verosimilitudes	,365	1	,546		
Estadístico exacto de Fisher				1,000	,484
Asociación lineal por lineal	,319	1	,572		
N de casos válidos	932				

a. 1 casillas (25,0%) tienen una frecuencia esperada inferior a 5. La frecuencia mínima esperada es 1,67.

b. Calculado sólo para una tabla de 2x2.

Estimación de riesgo

	Valor	Intervalo de confianza al 95%	
		Inferior	Superior
Razón de las ventajas para Apgar_1min_5 (1,00 / 2,00)	,557	,071	4,354
Para la cohorte GDM_DIAGNOSIS = SI	,594	,090	3,908
Para la cohorte GDM_DIAGNOSIS = NO	1,066	,897	1,267
N de casos válidos	932		

Tabla de contingencia

			GDM_DIAGNOSIS		Total
			SI	NO	
Apgar_5min_7	1,00	Recuento	0	5	5
		% dentro de GDM_DIAGNOSIS	,0%	,6%	,5%
	2,00	Recuento	130	797	927
		% dentro de GDM_DIAGNOSIS	100,0%	99,4%	99,5%

Total	Recuento	130	802	932
	% dentro de GDM_DIAGNOSIS	100,0%	100,0%	100,0%

Pruebas de chi-cuadrado

	Valor	gl	Sig. asintótica (bilateral)	Sig. exacta (bilateral)	Sig. exacta (unilateral)
Chi-cuadrado de Pearson	,815[a]	1	,367		
Corrección por continuidad[b]	,065	1	,798		
Razón de verosimilitudes	1,507	1	,220		
Estadístico exacto de Fisher				1,000	,471
Asociación lineal por lineal	,814	1	,367		
N de casos válidos	932				

a. 2 casillas (50,0%) tienen una frecuencia esperada inferior a 5. La frecuencia mínima esperada es ,70.

b. Calculado sólo para una tabla de 2x2.

Tabla de contingencia

			GDM_DIAGNOSIS		Total
			SI	NO	
NICU_STANCE	1,00	Recuento	0	9	9
		% dentro de GDM_DIAGNOSIS	,0%	1,1%	1,0%
	2,00	Recuento	130	793	923
		% dentro de GDM_DIAGNOSIS	100,0%	98,9%	99,0%
Total		Recuento	130	802	932
		% dentro de GDM_DIAGNOSIS	100,0%	100,0%	100,0%

Pruebas de chi-cuadrado

	Valor	gl	Sig. asintótica (bilateral)	Sig. exacta (bilateral)	Sig. exacta (unilateral)
Chi-cuadrado de Pearson	1,473[a]	1	,225		
Corrección por continuidad[b]	,533	1	,465		
Razón de verosimilitudes	2,718	1	,099		
Estadístico exacto de Fisher				,621	,257
Asociación lineal por lineal	1,471	1	,225		
N de casos válidos	932				

a. 1 casillas (25,0%) tienen una frecuencia esperada inferior a 5. La frecuencia mínima esperada es 1,26.

b. Calculado sólo para una tabla de 2x2.

Tabla de contingencia

			GDM_DIAGNOSIS		Total
			SI	NO	
OBSERVATION_STANCE	1,00	Recuento	2	17	19
		% dentro de GDM_DIAGNOSIS	1,5%	2,1%	2,0%
	2,00	Recuento	128	785	913
		% dentro de GDM_DIAGNOSIS	98,5%	97,9%	98,0%
Total		Recuento	130	802	932
		% dentro de GDM_DIAGNOSIS	100,0%	100,0%	100,0%

Pruebas de chi-cuadrado

	Valor	gl	Sig. asintótica (bilateral)	Sig. exacta (bilateral)	Sig. exacta (unilateral)
Chi-cuadrado de Pearson	,189[a]	1	,664		
Corrección por continuidad[b]	,010	1	,920		
Razón de verosimilitudes	,204	1	,652		
Estadístico exacto de Fisher				1,000	,492

Asociación lineal por lineal	,189	1	,664		
N de casos válidos	932				

a. 1 casillas (25,0%) tienen una frecuencia esperada inferior a 5. La frecuencia mínima esperada es 2,65.

b. Calculado sólo para una tabla de 2x2.

Estimación de riesgo

	Valor	Intervalo de confianza al 95%	
		Inferior	Superior
Razón de las ventajas para OBSERVATION_STANCE (1,00 / 2,00)	,722	,165	3,160
Para la cohorte GDM_DIAGNOSIS = SI	,751	,200	2,813
Para la cohorte GDM_DIAGNOSIS = NO	1,041	,890	1,217
N de casos válidos	932		

Tabla de contingencia

			GDM_DIAGNOSIS		Total
			SI	NO	
HYPOGLYCEMIA	SI	Recuento	1	7	8
		% dentro de GDM_DIAGNOSIS	,8%	,9%	,9%
	NO	Recuento	129	795	924
		% dentro de GDM_DIAGNOSIS	99,2%	99,1%	99,1%
Total		Recuento	130	802	932
		% dentro de GDM_DIAGNOSIS	100,0%	100,0%	100,0%

Pruebas de chi-cuadrado

	Valor	gl	Sig. asintótica (bilateral)	Sig. exacta (bilateral)	Sig. exacta (unilateral)
Chi-cuadrado de Pearson	,014[a]	1	,905		
Corrección por continuidad[b]	,000	1	1,000		
Razón de verosimilitudes	,015	1	,904		
Estadístico exacto de Fisher				1,000	,691
Asociación lineal por lineal	,014	1	,906		
N de casos válidos	932				

a. 1 casillas (25,0%) tienen una frecuencia esperada inferior a 5. La frecuencia mínima esperada es 1,12.

b. Calculado sólo para una tabla de 2x2.

Estimación de riesgo

	Valor	Intervalo de confianza al 95%	
		Inferior	Superior
Razón de las ventajas para HYPOGLYCEMIA (SI / NO)	,880	,107	7,215
Para la cohorte GDM_DIAGNOSIS = SI	,895	,142	5,640
Para la cohorte GDM_DIAGNOSIS = NO	1,017	,782	1,323
N de casos válidos	932		

Tabla de contingencia

			GDM_DIAGNOSIS		Total
			SI	NO	
RESPIRATORYDISTRESS	SI	Recuento	0	8	8
		% dentro de GDM_DIAGNOSIS	,0%	1,0%	,9%
	NO	Recuento	130	794	924
		% dentro de GDM_DIAGNOSIS	100,0%	99,0%	99,1%

Total	Recuento	130	802	932
	% dentro de GDM_DIAGNOSIS	100,0%	100,0%	100,0%

Pruebas de chi-cuadrado

	Valor	gl	Sig. asintótica (bilateral)	Sig. exacta (bilateral)	Sig. exacta (unilateral)
Chi-cuadrado de Pearson	1,308[a]	1	,253		
Corrección por continuidad[b]	,398	1	,528		
Razón de verosimilitudes	2,415	1	,120		
Estadístico exacto de Fisher				,609	,299
Asociación lineal por lineal	1,307	1	,253		
N de casos válidos	932				

a. 1 casillas (25,0%) tienen una frecuencia esperada inferior a 5. La frecuencia mínima esperada es 1,12.

b. Calculado sólo para una tabla de 2x2.

Tabla de contingencia

			GDM_DIAGNOSIS		Total
			SI	NO	
BRACHIALPLEXUS_INJURY	SI	Recuento	0	1	1
		% dentro de GDM_DIAGNOSIS	,0%	,1%	,1%
	NO	Recuento	130	801	931
		% dentro de GDM_DIAGNOSIS	100,0%	99,9%	99,9%
Total		Recuento	130	802	932
		% dentro de GDM_DIAGNOSIS	100,0%	100,0%	100,0%

Pruebas de chi-cuadrado

	Valor	gl	Sig. asintótica (bilateral)	Sig. exacta (bilateral)	Sig. exacta (unilateral)
Chi-cuadrado de Pearson	,162[a]	1	,687		
Corrección por continuidad[b]	,000	1	1,000		
Razón de verosimilitudes	,301	1	,583		
Estadístico exacto de Fisher				1,000	,861
Asociación lineal por lineal	,162	1	,687		
N de casos válidos	932				

a. 2 casillas (50,0%) tienen una frecuencia esperada inferior a 5. La frecuencia mínima esperada es ,14.

b. Calculado sólo para una tabla de 2x2.

Tabla de contingencia

			GDM_DIAGNOSIS		
			SI	NO	Total
HIPERBILIRRUBINEMIA	SI	Recuento	1	14	15
		% dentro de GDM_DIAGNOSIS	,8%	1,7%	1,6%
	NO	Recuento	129	788	917
		% dentro de GDM_DIAGNOSIS	99,2%	98,3%	98,4%
Total		Recuento	130	802	932
		% dentro de GDM_DIAGNOSIS	100,0%	100,0%	100,0%

Pruebas de chi-cuadrado

	Valor	gl	Sig. asintótica (bilateral)	Sig. exacta (bilateral)	Sig. exacta (unilateral)
Chi-cuadrado de Pearson	,673[a]	1	,412		
Corrección por continuidad[b]	,198	1	,656		

Razón de verosimilitudes	,809	1	,368		
Estadístico exacto de Fisher				,708	,358
Asociación lineal por lineal	,673	1	,412		
N de casos válidos	932				

a. 1 casillas (25,0%) tienen una frecuencia esperada inferior a 5. La frecuencia mínima esperada es 2,09.

b. Calculado sólo para una tabla de 2x2.

Estimación de riesgo

	Valor	Intervalo de confianza al 95%	
		Inferior	Superior
Razón de las ventajas para HIPERBILIRRUBINEMIA (SI / NO)	,436	,057	3,346
Para la cohorte GDM_DIAGNOSIS = SI	,474	,071	3,169
Para la cohorte GDM_DIAGNOSIS = NO	1,086	,946	1,247
N de casos válidos	932		

Tabla de contingencia

			LGA_4000_4500		Total
			1,00	2,00	
EWG_VS_ADEQUATE	1,00	Recuento	5	352	357
		% dentro de LGA_4000_4500	16,7%	48,0%	46,8%
	2,00	Recuento	25	381	406
		% dentro de LGA_4000_4500	83,3%	52,0%	53,2%
Total		Recuento	30	733	763
		% dentro de LGA_4000_4500	100,0%	100,0%	100,0%

Pruebas de chi-cuadrado

	Valor	gl	Sig. asintótica (bilateral)	Sig. exacta (bilateral)	Sig. exacta (unilateral)
Chi-cuadrado de Pearson	11,381[a]	1	,001		
Corrección por continuidad[b]	10,156	1	,001		
Razón de verosimilitudes	12,554	1	,000		
Estadístico exacto de Fisher				,001	,000
Asociación lineal por lineal	11,366	1	,001		
N de casos válidos	763				

a. 0 casillas (,0%) tienen una frecuencia esperada inferior a 5. La frecuencia mínima esperada es 14,04.

b. Calculado sólo para una tabla de 2x2.

Estimación de riesgo

	Valor	Intervalo de confianza al 95%	
		Inferior	Superior
Razón de las ventajas para EWG_VS_ADEQUATE (1,00 / 2,00)	,216	,082	,572
Para la cohorte LGA_4000_4500 = 1,00	,227	,088	,588
Para la cohorte LGA_4000_4500 = 2,00	1,051	1,022	1,080
N de casos válidos	763		

Estadísticos de grupo

	GDM_DIAGNOSIS	N	Media	Desviación típ.	Error típ. de la media
AGE	SI	70	35,17	4,260	,509

	NO	314	32,71	4,876	,275
kg	SI	70	60,9057	11,14206	1,33173
	NO	312	59,5341	8,81650	,49914
BMIPREGESTATIONAL	SI	70	23,3257	4,18483	,50018
	NO	311	22,3821	3,17434	,18000
COMPUTE BMIPREGESTACIONAL=Pesopregestacional / (TALLAmetros * TALLAmetros)	SI	70	23,3257	4,18483	,50018
	NO	311	22,3821	3,17434	,18000
COMPUTE GananciaPesoSG38=PESO_SG36 - Pesopregestacional	SI	65	9,9862	5,70895	,70811
	NO	262	12,5731	5,23514	,32343
KILOGRAMOS	SI	48	63,5979	9,19737	1,32753
	NO	220	64,2368	10,70558	,72177
BMI_POSTPARTO	SI	48	24,5752	4,14392	,59812
	NO	220	24,1571	4,03018	,27171
COMPUTE GANANCIASG12_POSTPARTO = PESOPostP - PESO_SG12	SI	48	1,1833	3,70838	,53526
	NO	218	3,4486	4,82374	,32671
CM	SI	45	84,7800	8,61325	1,28399
	NO	203	83,8808	9,07365	,63685
SBP_AFTERDELIVERY12_14W	SI	44	110,65	11,895	1,793
	NO	203	110,45	12,899	,905
DBP_AFTERDELIVERY_12_14w	SI	44	73,68	9,557	1,441
	NO	203	72,53	10,459	,734
FBG_12WAFTERDELIVERY	SI	70	85,96	7,569	,905
	NO	314	84,26	7,632	,431
HBA1C_12WsAFTERDELIVERY	SI	67	5,330	,2505	,0306
	NO	267	5,204	,2836	,0174
COL_12WsAFTERDELIVERY	SI	69	204,57	41,849	5,038
	NO	312	194,60	38,214	2,163

HDL_12WsAFTERDELIVERY	SI	17	67,8235	12,45108	3,01983
	NO	65	62,6154	15,27528	1,89467
LDL_12WsAFTERDELIVERY	SI	17	129,7059	44,11718	10,69999
	NO	65	120,1626	31,74704	3,93774
TrRIG_12WsAFTERDELIVERY	SI	68	79,68	51,789	6,280
	NO	310	79,54	41,723	2,370
APO_12WsAFTERDELIVERY	SI	16	97,2063	28,27789	7,06947
	NO	49	89,4106	28,25813	4,03688
INSULINA_12WsAFTERDELIVERY	SI	62	5,6013	3,95535	,50233
	NO	260	6,6378	5,63188	,34927
HOMA_12WsAFTERDELIVERY	SI	47	1,6106	1,64240	,23957
	NO	225	1,5801	1,33904	,08927
TSH_12WsAFTERDELIVERY	SI	70	2,1496	1,81392	,21681
	NO	300	2,1690	5,02487	,29011
T4L_12WsAFTERDELIVERY	SI	70	7,9851	1,22722	,14668
	NO	300	8,3153	3,28968	,18993
PREG_NUTRITIONSCORE	SI	66	,7576	3,01310	,37089
	NO	299	,6154	2,98698	,17274
NUTRITION_SCORE_36GW	SI	47	8,0851	2,99151	,43636
	NO	209	3,7416	3,03509	,20994
COMPUTE	SI	63	4,4603	1,40075	,17648

SG12_MEDDIET_SCORE= SG12_ACEITEOLIVAMEDDIET + SG12_MLACEITEDIA_MEDDIET + SG12_VERDURAS_MEDDIET + SG12_FRUTAS_MEDDIET + SG12_CARNEPROCESADA_MEDDIET + SG12_MANTEQUILLA_MEDDIET + SG12_BEBIDASAZUCARADAS_MEDDIET + SG12_ALCOHOL_MEDDIET + SG12_LEGUMBRES_ME	NO	280	4,4357	1,69634	,10138
COMPUTE	SI	33	7,8485	2,18119	,37970

SG36_MEDDIET_SCORE= SG36_ACEITEOLIVAMEDDIET + SG36_MLACEITEDIA_MEDDIET + SG36_VERDURAS_MEDDIET + SG36_FRUTAS_MEDDIET + SG36_CARNEPROCESADA_MEDDIET + SG36_MANTEQUILLA_MEDDIET + SG36_BEBIDASAZUCARADAS_MEDDIET + SG36_ALCOHOL_MEDDIET + SG36_LEGUMBRES_ME	NO	135	5,3630	1,79388	,15439

Prueba de muestras independientes

		Prueba de Levene para la igualdad de varianzas		Prueba T para la igualdad de medias						
									95% Intervalo de confianza pa la diferencia	
		F	Sig.	t	gl	Sig. (bilateral)	Diferencia de medias	Error típ. de la diferencia	Inferior	Superior
AGE	Se han asumido varianzas iguales	,758	,385	3,909	382	,000	2,464	,631	1,225	3,7
	No se han asumido varianzas iguales			4,258	113,069	,000	2,464	,579	1,318	3,6
kg	Se han asumido varianzas iguales	,722	,396	1,117	380	,265	1,37158	1,22759	-1,04215	3,785
	No se han asumido varianzas iguales			,964	89,356	,337	1,37158	1,42220	-1,45414	4,197

BMIPREGESTATIONAL	Se han asumido varianzas iguales	2,814	,094	2,110	379	,036	,94364	,44726	,06421	1,823
	No se han asumido varianzas iguales			1,775	87,702	,079	,94364	,53158	-,11282	2,000
COMPUTE BMIPREGESTACIONAL=Pesopregestacional / (TALLAmetros * TALLAmetros)	Se han asumido varianzas iguales	2,814	,094	2,110	379	,036	,94364	,44726	,06421	1,823
	No se han asumido varianzas iguales			1,775	87,702	,079	,94364	,53158	-,11282	2,000
COMPUTE GananciaPesoSG38=PESO_SG36 - Pesopregestacional	Se han asumido varianzas iguales	,616	,433	-3,501	325	,001	-2,58694	,73882	-4,04041	-1,133
	No se han asumido varianzas iguales			-3,323	92,502	,001	-2,58694	,77847	-4,13294	-1,040
KILOGRAMOS	Se han asumido varianzas iguales	1,542	,215	-,384	266	,702	-,63890	1,66555	-3,91823	2,640
	No se han asumido varianzas iguales			-,423	77,441	,674	-,63890	1,51105	-3,64752	2,369
BMI_POSTPARTO	Se han asumido varianzas iguales	,727	,394	,648	266	,518	,41808	,64528	-,85242	1,688
	No se han asumido varianzas iguales			,636	67,781	,527	,41808	,65695	-,89291	1,729
COMPUTE GANANCIASG12_POSTPARTO = PESOPostP - PESO_SG12	Se han asumido varianzas iguales	,658	,418	-3,059	264	,002	-2,26529	,74056	-3,72345	-,807
	No se han asumido varianzas iguales			-3,612	85,959	,001	-2,26529	,62709	-3,51191	-1,018
CM	Se han asumido varianzas iguales	,331	,565	,607	246	,545	,89921	1,48176	-2,01934	3,817
	No se han asumido varianzas iguales			,627	67,423	,533	,89921	1,43325	-1,96123	3,759
SBP_AFTERDELIVERY12_14W	Se han asumido varianzas iguales	,474	,492	,097	245	,923	,205	2,117	-3,964	4,3
	No se han asumido varianzas iguales			,102	66,791	,919	,205	2,009	-3,804	4,2

DBP_AFTERDELIVERY_12_14w	Se han asumido varianzas iguales	,117	,733	,674	245	,501	1,155	1,714	-2,221	4,5
	No se han asumido varianzas iguales			,714	67,259	,478	1,155	1,617	-2,073	4,3
FBG_12WAFTERDELIVERY	Se han asumido varianzas iguales	,180	,672	1,684	382	,093	1,696	1,007	-,284	3,6
	No se han asumido varianzas iguales			1,693	102,660	,094	1,696	1,002	-,291	3,6
HBA1C_12WsAFTERDELIVERY	Se han asumido varianzas iguales	,542	,462	3,328	332	,001	,1261	,0379	,0516	,20
	No se han asumido varianzas iguales			3,585	112,417	,001	,1261	,0352	,0564	,19
COL_12WsAFTERDELIVERY	Se han asumido varianzas iguales	1,008	,316	1,927	379	,055	9,970	5,174	-,203	20,1
	No se han asumido varianzas iguales			1,818	94,688	,072	9,970	5,483	-,916	20,8
HDL_12WsAFTERDELIVERY	Se han asumido varianzas iguales	,023	,881	1,296	80	,199	5,20814	4,01909	-2,79011	13,206
	No se han asumido varianzas iguales			1,461	29,917	,154	5,20814	3,56499	-2,07338	12,489
LDL_12WsAFTERDELIVERY	Se han asumido varianzas iguales	2,903	,092	1,013	80	,314	9,54327	9,41916	-9,20146	28,288
	No se han asumido varianzas iguales			,837	20,533	,412	9,54327	11,40156	-14,20043	33,286
TrRIG_12WsAFTERDELIVERY	Se han asumido varianzas iguales	,735	,392	,023	376	,981	,136	5,850	-11,367	11,6
	No se han asumido varianzas iguales			,020	87,053	,984	,136	6,713	-13,206	13,4
APO_12WsAFTERDELIVERY	Se han asumido varianzas iguales	,125	,725	,958	63	,342	7,79564	8,13794	-8,46674	24,058
	No se han asumido varianzas iguales			,958	25,529	,347	7,79564	8,14087	-8,95321	24,544
INSULINA_12WsAFTERDELIVERY	Se han asumido varianzas iguales	2,693	,102	-1,370	320	,172	-1,03648	,75655	-2,52492	,451

	No se han asumido varianzas iguales			-1,694	127,235	,093	-1,03648	,61182	-2,24715	,174
HOMA_12WsAFTERDELIVERY	Se han asumido varianzas iguales	,004	,952	,137	270	,892	,03055	,22379	-,41004	,471
	No se han asumido varianzas iguales			,119	59,426	,905	,03055	,25566	-,48095	,542
TSH_12WsAFTERDELIVERY	Se han asumido varianzas iguales	,088	,767	-,032	368	,975	-,01940	,61019	-1,21929	1,180
	No se han asumido varianzas iguales			-,054	308,827	,957	-,01940	,36217	-,73204	,693
T4L_12WsAFTERDELIVERY	Se han asumido varianzas iguales	2,013	,157	-,826	368	,409	-,33019	,39987	-1,11651	,456
	No se han asumido varianzas iguales			-1,376	299,834	,170	-,33019	,23998	-,80244	,142
PREG_NUTRITIONSCORE	Se han asumido varianzas iguales	,137	,711	,349	363	,727	,14219	,40687	-,65792	,942
	No se han asumido varianzas iguales			,348	95,281	,729	,14219	,40914	-,67003	,954
NUTRITION_SCORE_36GW	Se han asumido varianzas iguales	,245	,621	8,888	254	,000	4,34348	,48870	3,38105	5,305
	No se han asumido varianzas iguales			8,970	68,944	,000	4,34348	,48423	3,37745	5,309
COMPUTE SG12_MEDDIET_SCORE=	Se han asumido varianzas iguales	1,938	,165	,107	341	,915	,02460	,22960	-,42701	,476

SG12_ACEITEOLIVAMEDDIET + SG12_MLACEITEDIA_MEDDIET + SG12_VERDURAS_MEDDIET + SG12_FRUTAS_MEDDIET + SG12_CARNEPROCESADA_MEDDIET + SG12_MANTEQUILLA_MEDDIET + SG12_BEBIDASAZUCARADAS_MEDDIET + SG12_ALCOHOL_MEDDIET + SG12_LEGUMBRES_ME	No se han asumido varianzas iguales			,121	107,077	,904	,02460	,20352	-,37885	,428
COMPUTE SG36_MEDDIET_SCORE=	Se han asumido varianzas iguales	3,197	,076	6,827	166	,000	2,48552	,36407	1,76672	3,204

SG36_ACEITEOLIVAMEDDIET + SG36_MLACEITEDIA_MEDDIET + SG36_VERDURAS_MEDDIET + SG36_FRUTAS_MEDDIET + SG36_CARNEPROCESADA_MEDDIET + SG36_MANTEQUILLA_MEDDIET + SG36_BEBIDASAZUCARADAS_MEDDIET + SG36_ALCOHOL_MEDDIET + SG36_LEGUMBRES_ME	No se han asumido varianzas iguales			6,064	43,175	,000	2,48552	,40989	1,65901	3,312
